# Assessment of cytotoxic and antimicrobial activities of two components of *Cymbopogon citratus* essential oil

**DOI:** 10.4317/jced.56863

**Published:** 2020-08-01

**Authors:** Carolina Chaves-Quirós, Johnatan-Stiven Usuga-Usuga, Sandra-Milena Morales-Uchima, Adriana-Patricia Tofiño-Rivera, Sergio-Iván Tobón-Arroyave, María-Cecilia Martínez-Pabón

**Affiliations:** 1 Graduate Periodontics Resident. Department of Periodontics, Faculty of Dentistry, University of Antioquia. Medellín, Colombia; 2 MSc Microbiology and Bioanalysis. Laboratory of Oral Microbiology, Faculty of Dentistry, University of Antioquia. Medellín, Colombia; 3 PhD Agrarian Sciences. Motilonia Research Center, Colombian Corporation for Agricultural Research (Agrosavia), Cesar, Colombia; 4 Specialist in Stomatology and Oral Surgery. Laboratory of Immunodetection and Bioanalysis, Faculty of Dentistry, University of Antioquia. Medellín, Colombia; 5 MSc Microbiology. Laboratory of Oral Microbiology, Faculty of Dentistry, University of Antioquia. Medellín, Colombia

## Abstract

**Background:**

There is a continuing search for compuounds to improve the chemical plaque inhibitory action of oral hygiene products. Although the antibacterial effects of chlorhexidine (CHX) and essential oils components, citral/myrcene, have been described, there is contradictory information regarding their cytotoxic effects in host tissues. This study aimed to evaluate the cytotoxic activity of the major components of the oil *C. citratus*, citral and myrcene on human periodontal ligament fibroblast (HPLF) cultures and their antimicrobial effect on different bacterial species present in supragingival biofilm.

**Material and Methods:**

Cytotoxicity of the compounds to HPLF was determined by MTT assay. Antimicrobial activity was tested against reference strains of *Enterococcus faecalis*, *Streptococcus mutans* and *Lactobacillus rhamnosus* and for *S. mutans* clinical strains by broth microdilution assay. One-way analysis of variance (ANOVA) with Games-Howell post-hoc multiple comparison or unpaired t tests were used for inter- and intragroup comparisons.

**Results:**

Overall, all of the compounds under study showed a cytotoxic effect to HPLF which varied in a dose-dependant manner. Whilst myrcene did not show bacteriostatic activity at tested concentrations, both citral and CHX exhibited bacteriostatic/bactericidal effects to all strains at specific concentrations, being CHX most effective to inhibit bacterial growth at lower concentrations than what observed for citral.

**Conclusions:**

Based on these findings, it would possible to conclude that whereas myrcene might be ineffective to control bacterial growth, citral could have a promising antimicrobial activity against dental colonizers with low cytotoxicity, and may be useful for preventing the onset and progression of oral diseases.

** Key words:**Antimicrobial activity, citral, cytotoxicity, chlorhexidine, myrcene.

## Introduction

Increasing evidence emphasizes the effectiveness of mechanical and chemical removal of biofilms in order to prevent the development of caries and periodontal diseases (1,2). Among chemical agents, several compounds including triclosan, sodium fluoride, chlorhexidine (CHX), essential oils (EOs), and other antiseptic solutions had been widely examined in different settings (3-5). Although CHX is considered the gold standard antimicrobial agent not only because of its fungicidal, bactericidal, and bacteriostatic effects, which are dose- and species-dependent (6), but also to the high substantivity and lack of bacterial resistance (7), frequently local adverse effects including teeth pigmentation, formation of supragingival calculi, dysgeusia, and irritation of oral mucosa have been found (8). Moreover, other harmful side effects such as cytotoxicity, induction of apoptosis on fibroblasts, distrurbance of cell proliferation, reduction in the production of collagen and non-collagen proteins (9), and impaired wound healing (10) have been also described.

In order to avoid the side effects of synthetic products, natural products, such as pure compounds or standardized plant extracts, have provided opportunities as new drugs for control of biofilm formation due to their chemical diversity (11). In this sense, previous researches have demonstrated the antimicrobial activity of the essential oil *Cymbopogon citratus* on *Streptococcus mutans* planktonic cultures (12,13) and *S. mutans* biofilms (14-16). This essential oil of *C. citratus* has three main components: alpha citral (geranial), beta citral (neral) and myrcene. Whereas, it has been shown that both alpha and beta citral are antimicrobial for gram-positive and gram-negative bacteria and fungistatic for *Candida* spp. (17), myrcene has not demonstrated antimicrobial activity (17,18). Considering not only that there is contradictory information regarding cytotoxic effects of essential oils components, but also that the loss of balance between the species that make up the dental biofilm is essential to prevent the onset and progression of oral diseases, this study aimed to evaluate the cytotoxic activity of the major components of the oil *C. citratus*, citral and myrcene on human periodontal ligament fibroblast (HPLF) cultures and their antimicrobial effect on different bacterial species present in supragingival biofilm.

## Material and Methods

This experimental study was conducted in the Laboratory of Oral Microbiology of Faculty of Dentistry of the University of Antioquia in Medellín (Colombia) and ethical approval was obtained from the Institutional Ethics Committee for Human Studies (reference code 06-2018).

-Cells, experimental conditions, and MTT cytotoxic assay

The test was performed following previously described procedures (19,20). Myrcene (Sigma-Aldrich®, Dorset, UK), citral (geranial and neral mixture, Alfa Aesar®, Heysham, UK), and CHX (Sigma-Aldrich®) were evaluated in HPLF monolayer cultures kindly provided by the Dental Research Center of Pontifical Javerian University. These HPLFs were seeded in 96-well microtiter plates at a density of 5.00E+4 cells/well and maintained under suiTable culture conditions. Once the cells were attached, triplicate treatments of the compounds diluted in 1% dimethyl sulphoxide (DMSO) were added. For myrcene and citral the concentrations used were 1.00E-6, 1.00E-5, 1.00E-4, 1.00E-3, 1.00E-2, 5.00E-1, 1.00E+0 and 2.00E+0 %w/v, whilst 1.00E-6, 1.00E-5, 1.00E-4, 1.00E-3, 1.00E-2 %w/v were used as concentrations for CHX. Also, cells cultured with 50% hydrogen peroxide (H2O2) solution were used as cell death control and untreated cells as control of viability. After 24-h of exposure to the compounds, 10 μL of MTT reagent (Sigma-Aldrich®) was added to 5 mg/mL of each well of the microtiter plate in order to obtain a final concentration in the well of 0.5 mg/mL. Plates were incubated for 4-h at 37 °C in darkness. After incubation period, the MTT solution was removed and the formazan crystals obtained were solubilized in 100 μL in a solution of isopropanol-HCl (Merck KGaA. Darmstadt, Germany) 0.04 M. Thereafter, the absorbance was measured by using a enzyme linked immunosorbent assay (ELISA) reader (Multiskan FC®, Thermo Scientific, Waltham, MA, USA) set at 571 nm. The GraphPad Prism 6 software (GraphPad Prism Inc., San Diego, CA, USA) was used to calculate the 50% inhibitory concentration (IC50), which was defined as the concentration of the compounds that reduced the viability of the treated cells by 50% with respect to untreated cells (21).

-Bacterial culture, strains, and growth conditions

Reference strains used were *Enterococcus faecalis* ATCC 29212, *S. mutans* ATCC 25175, *S. mutans* ATCC 35668, *S. mutans* ATCC UA159, and *Lactobacillus rhamnosus* ATCC 53103. Additionaly, eight randomly selected clinical isolates of *S. mutans* obtained from schoolchildren with white spot and cavitated caries lesions from a previous study (22) were included. For determination of the minimum inhibitory concentration (MIC), agar dilution assays were performed following the methodology given in a previous study (15). Compounds under study were serially diluted in 2-fold steps and then 1 mL of each dilution was added to 18 mL of brain heart infusion (BHI) agar and mixed thoroughly before pouring. Two control plates containing no compound were used to control sterility and growth. Agar plates were used within the first 24-h after its preparation. A standardized inoculum was prepared for each strain tested. After overnight incubation in BHI broth at 37°C, bacteria were collected by and suspended in sterile BHI broth. Turbidity was adjusted to 0.5 McFarland standard by adding BHI broth, up to a cell density of ~1.00E+8 CFU/mL. Subsequently, this suspension was further diluted in 10-fold step and 2 μL of diluted broth culture, providing a final inoculum of approximately 1.00E+4 CFU/mL, which was spot-inoculated onto agar plates. Inoculated plates were incubated for triplicate during 24-h at 37°C before determination of MICs. MIC was defined as the lowest concentration at which there was no visible growth.

In addition to the former, the minimum bactericide concentration (MBC), defined as the lowest concentration of the essential oil components at which incubated microorganism was completely killed, was determined using the microdilution reference method (15) with modifications. Concentrations ranging from 2.50E-1 to 2.00E+0 mg/mL of the essential oil component that demonstrated inhibitory effects during MIC determinations were obtained in culture medium (BHI broth). After the inoculation of standardized suspensions with 10 μL of the inoculum, the microtiter plates were incubated for 24-h at 37°C. The suspensions were diluted as described for MIC and 10 μL were spot-inoculated onto BHI agar plates to evaluate bacterial growth. A 0.12% CHX solution (Sigma-Aldrich®) was used as the positive control, culture media without chemical inhibitors were used as growth controls, and culture media without inoculum were used as sterility control.

-Statistical methods and data analysis

Data management and statistical analysis were performed by using standard statistical software (SPSS 25.0®, IBM, Armonk, NY). The normality of distribution of each variable was tested via the Shapiro-Wilk test. Because the data showed normal distributions, the variables were analyzed using parametric methods and the data were expressed as mean ± SD. For inter- and intragroup comparisons, differences on viability percentage regarding the compound concentrations and the controls were analyzed via one-way analysis of variance (ANOVA) with Games-Howell *post-hoc* multiple comparison tests or unpaired t tests when indicated. All tests were two-sided and statistical significance was assumed at a *P*-value <0.05.

## Results

-MTT cytotoxic assay

The MTT test results on cultured HPLF are depicted in Table 1. As can be seen from this Table all of the compounds under study showed a cytotoxic effect which varied in a dose-dependant manner. It was noticeable that whilst CHX was significantly less cytotoxic at a concentration of 1.00E-5 % w/v than citral and myrcene (*P* <0.05, Games-Howell *post-hoc* multiple comparison test), to the greater concentrations (1.00E-3 and 1.00E-2 % w/v) a significant higher cell proliferation (*P* <0.05) was observed after cultivation with citral and myrcene. Also, it could be observed that myrcene had only a more significant cytotoxic effect than citral (*P* <0.05, unpaired t test) at a concentration of 2.00E+0 % w/v. It was also noteworthy, that cytotoxic effects of citral and myrcene were significantly greater at concentrations ranging from 5.00E-1 to 2.00E+0 % w/v than those observed for 50% hydrogen peroxide (*P* <0.05, Games-Howell post-hoc multiple comparison test). In regards to intra-group comparisons, whilst CHX demostrated a greater cytotoxic effect at 1.00E-3 and 1.00E-2 % w/v than those observed at higher concentrations, the highest cytotoxic effects of citral and myrcene were observed at concentrations varying among 5.00E-1 to 2.00E+0 % w/v ( all *P* <0.05). Alternatively, it was remarkable that the concentration of compounds required to produce 50% reduction in the viability of HPLF (IC50) was lower for CHX (1.3E-3% w/v) in comparison with those values observed for citral (4.7E-1% w/v) and myrcene (4.3E-1% w/v).

**Table 1:**
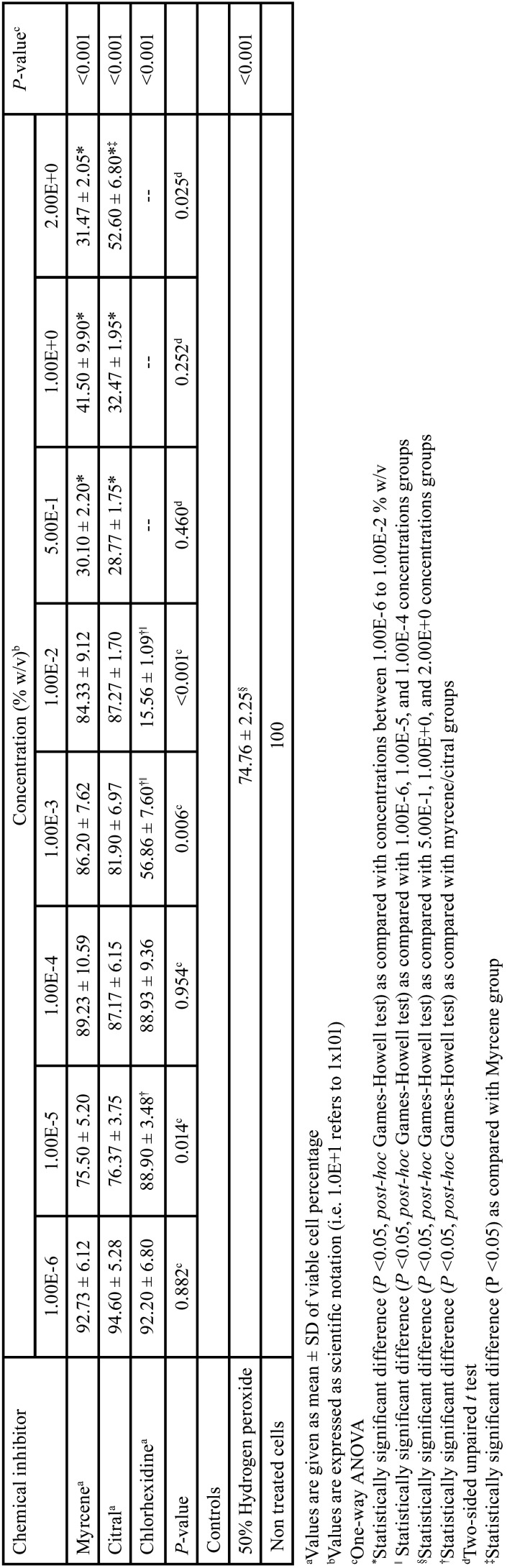
Inter- and intragroup comparisons of viability percentage of periodontal ligament fibroblasts according to the concentrations of different chemical inhibitors.

-Determination of MIC and MBC of compounds under study on selected bacterial strains

The results of MIC bacterial-viability tests are displayed in Table 2. The visualized colonies on the agar plates indicated that whilst myrcene did not show bacteriostatic activity neither against *E. faecalis*, *S. mutans*, nor *L. ramnosus* at tested concentrations, both citral and CHX exhibited bacteriostatic effects to all strains at specific concentrations, including those of clinical isolates from schoolchildren, being CHX most effective to inhibit bacterial growth at lower concentrations than what observed for citral. As myrcene did not show bacteriostatic activity, only citral was evaluated for MBC determination. Accordingly, although it could be observed that citral showed bactericidal activity against all tested strains (Table 3) at a concentration of 1.00E+0 mg/mL, some clinical strains showed no growth at concentrations of 0.50E+0 mg/mL.

**Table 2:**
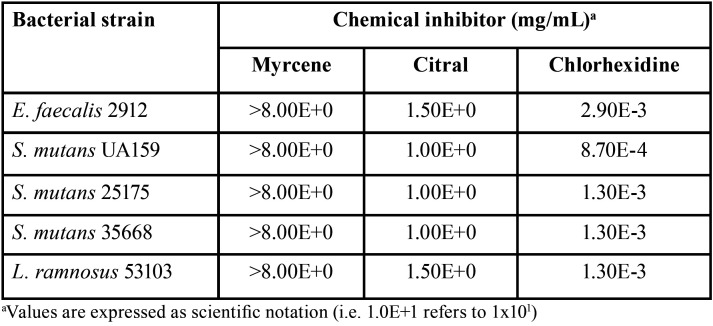
Minimal inhibitory concentration (MIC) of chemical inhibitors under study regarding bacterial strains.

**Table 3:**
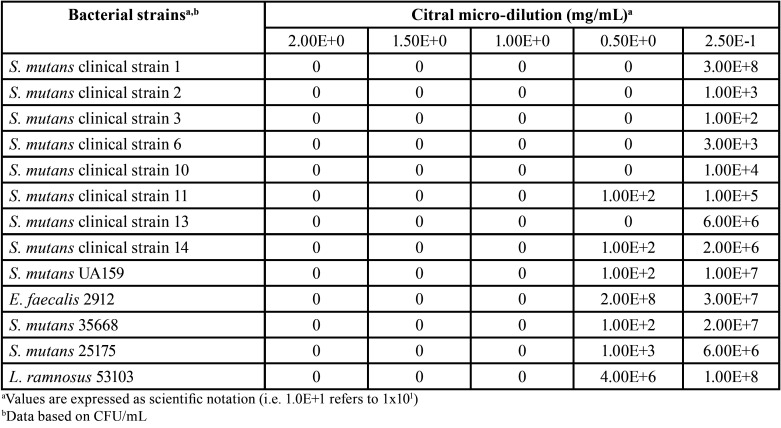
Minimum bactericide concentration (MBC) of citral regarding *S. mutans* clinical and reference strains.

## Discussion

Different studies have demonstrated the effectiveness of mouthwashes containing antimicrobial active ingredients such as chlorhexidine and essential oils in controlling both supra- and subgingival plaque when used adjunctively to mechanical oral hygiene regimens (6-8,12,14-17,23). Even so, there are concerns that these products are harmful to human oral cells (24,25). Owing these facts, it is important to generate new insights that supports the day-to-day use of essential oils components as therapeutic alternative in oral health.

It is well known that CHX is toxic, even in low concentrations, for different cell types including epithelial cells, gingival fibroblasts, neutrophils, macrophages, red blood cells, and fibroblasts in culture (26,27). Likewise, it has been previously demonstrated that even topical application of CHX can result in its penetration through the epithelial barrier leading to tissue damage (26). In agreement to the former, in the present study, CHX showed significantly higher cytotoxic effects on HPLF cultures as the concentration increased. The present findings might parallel, at least partially, those reported by others (3), which found that CHX induced apoptosis and/or necrosis of cultured fibroblasts very likely via endoplasmic reticulum stress in a concentration-dependent manner. On the contrary, while some researchers suggest that essential oils or their components do not pose a risk of producing cell death or toxicity (14,16,28), others have shown that depending on type and concentration, they exhibit cytotoxic effects on living cells but are usually non-genotoxic (15,29). The data presented herein confirm although that both citral and myrcene had cytotoxic effects even at low concentrations, the magnitude of toxicity increased with the increase in concentration of the compounds. In consistence with the aforementioned, it has been postulated that such effects are due to prooxidant mechanims on the cellular level. Being lipophiles, these compounds may pass through the cytoplasmic membrane, disrupt the structure of their different layers of polysaccharides, fatty acids and phospholipids and rendering them more permeable, so that cytotoxicity appears to include such membrane damage (29,30). In eukaryotic cells, essential oils components can also provoke depolarization of the mitochondrial membranes. They change the fluidity of membranes, which become abnormally permeable resulting in leakage of radicals, cytochrome C, calcium ions and proteins, as in the case of oxidative stress and bioenergetic failure (29). Permeabilization of outer and inner mitochondrial membranes leads to cell death by apoptosis and necrosis (31,32).

It is well known that CHX acts as a detergent by absorbing onto the cell wall of prokaryotes and causing leakage of intracellular components (33). However, although the antibacterial activity of essential oils and their compounds used as mouthrinses has been extensively studied (14-17,23), the mechanisms which explain it, have not been fully described. Given that both citral and myrcene are phenols and terpenes in nature, it has been postulated that their mode of action might be similar to that of other phenolic compounds (34). In this sense it is thought that phenolic compounds not only attack cell wall and cell membrane, thereby destroying its permeability and releasing intracellular constituents such as ribose, Na, and glutamate. Also they interfere with membrane functions such as electron transport, nutrient uptake, protein and nucleic acid synthesis and enzyme activity (29,34). The present results concur with previous studies that have shown not only that different microorganisms may exhibit different susceptivity to the same essential oil compound or CHX (16) but also that myrcene lacks of antimicrobial activity (17,18). Likewise, both MIC and MBC values detected in this work showed similar concentration ranges to those previouly described (15). As a final point, taking into account that MIC and MBC of citral against tested bacterial strains is even lower than that of commercially available CHX (1.20E+0 mg/mL), this compound might be defined as bactericidal rather than bacteriostatic (35). Furthermore, given that MIC and MBC values on reference strains (1.00E+0 mg/mL) were either higher or similar to those of clinical isolates (0.50E+0 to 1.00E+0 mg/mL), it would possible to assume that citral possess antimicrobial clinical activity with no evidence of resistance. Alternatively, its lower cytotoxic activity indicates that this might be an interesting compound for further development as adjunct to mechanical oral biofilm control.

The main limitation of this study was that bacterial strains tested may not represent the diversity of the microorganisms present within the oral microenvironment. Therefore, a more complete understanding regarding the antimicrobial effects of these compounds can certainly be obtained from studies that combine both the analysis of cultivated bacteria as well as that of bacteria directly obtained from individuals with different dental and periodontal status.

Taken altogether, based on the current data, it would possible to conclude that whereas myrcene might be ineffective to control bacterial growth, citral could have a promising antimicrobial activity against dental colonizers with low cytotoxicity, and may be useful for preventing the onset and progression of oral diseases.
